# Accuracy of ECG chest electrode placements by paramedics: an observational study

**DOI:** 10.29045/14784726.2019.12.4.3.51

**Published:** 2019-12-01

**Authors:** Pete Gregory, Stephen Lodge, Tim Kilner, Suzy Paget

**Affiliations:** University of Wolverhampton: ORCID iD: https://orcid.org/0000-0001-9845-0920; University of Wolverhampton; University of Worcester; University of Wolverhampton

## Abstract

**Introduction:**

The main aim of this study was to ascertain the accuracy of the chest lead placements by registered paramedics.

**Methods:**

Registered paramedics who attended the Emergency Services Show in Birmingham in September 2018 were invited to participate in this observational study. Participants were asked to place the chest electrodes on a human male model in accordance with their current practice. Correct positioning was determined against the Society for Cardiological Science and Technology’s 2017 clinical guidelines for recording a standard 12-lead electrocardiogram, with a tolerance of 19 mm being deemed acceptable based upon previous studies. Participants were also asked to indicate what they believed to be the correct positions on an anatomical diagram, and to describe the landmarks and positions in writing.

**Results:**

A total of 52 eligible participants completed the study. Measurement of electrode placement in the craniocaudal and mediolateral planes showed a high level of inaccuracy, with 3/52 (5.8%) participants able to accurately place all chest leads. In leads V1–V3, the majority of incorrect placements were related to vertical displacement, with most participants able to identify the correct horizontal position. In V4, the tendency was to place the lead too low and to the left of the pre-determined position, while V5 tended to be below the expected positioning but in the correct horizontal alignment. There was a less defined pattern of error in V6, although vertical displacement was more likely than horizontal displacement. Only 1.9% of participants were able to correctly label the diagram and 1.9% were correctly able to write down the landmarks and correct positions.

**Conclusion:**

Our study identified a high level of variation in the placement of chest ECG electrodes, which could alter the morphology of the ECG. There was also a high degree of inaccuracy in the written components of the study, which suggests that underpinning knowledge is likely to be a major factor behind this variation. From a patient safety perspective, we would advocate that paramedics leave the chest electrodes in situ to allow hospital staff to assess the accuracy of the placements. Further consideration needs to be given to initial and ongoing training of ECG electrode placement to improve performance.

